# Nutritional Status among 6-59 Months Children in a Selected Community of a VDC

**DOI:** 10.31729/jnma.4276

**Published:** 2019-04-30

**Authors:** Basudev Bhattarai, K.C. Yadav, Dip Narayan Thakur, Kiran Pandey

**Affiliations:** 1 Department of Nutrition and Dietetics, Central Campus of Technology, Tribhuvan University, Dharan, Nepal; 2 Department of Food Technology, Central Campus of Technology, Tribhuvan University, Dharan, Nepal; 3 Department of Community Medicine and Public Health, Institute of Medicine, Tribhuvan University, Kathmandu, Nepal; 4 College of Medical Sciences, Kathmandu University, Bharatpur, Chitwan, Nepal

**Keywords:** *malnutrition*, *nutrition*, *undernutrition*

## Abstract

**Introduction:**

Adequate nutrition is essential for proper growth and development of the child. We conducted a cross-sectional descriptive study to find the prevalence of nutritional status of 6-59 month old Tharu children in Duruwa of Dang District. This study also deliberates the socioeconomic and demographic character, maternal and child character and child feeding status of the Tharu community children.

**Methods:**

This study was a descriptive cross sectional study and was done between February and March, 2017 in Tharu community of Duruwa, Dang. Total 189 children of age group 6-59 months were selected by using systematic random sampling technique; anthropometric measurements were performed to find the nutritional status of children. Pre-coded questionnaire was used to collect information from caretaker. WHO Anthro version 3.2.2 and SPSS version 20 were used to analyze data.

**Results:**

Prevalence of wasting, stunting, and underweight were 25 (13.2%), 51 (27%), and 40 (21.2%) respectively. The prevalence of stunting was seen more in females than in males while underweight was higher among males, whereas wasting was very similar in both sexes.

**Conclusions:**

The findings showed that there is still high prevalence of malnutrition in Tharu children of Duruwa so monitoring of nutrition status in Tharu children should be taken seriously and needs to be addressed.

## INTRODUCTION

Adequate nutrition is essential in children for their good mental and physical development.^1^ Thirty-six percent of children under five in Nepal are stunted, 10% are wasted and 27% are underweight.^2^

Tharu community are deprived due to poor health, low economic status, lack of decision making on developmental works, poor value for their labor works, less rights in natural resources, no market access, and other various social factors.^3^ There have been no prior studies assessing the nutritional status of Tharu's children. In this regard, this study provides nutritional status of Tharu's children and factors associated with it. This study would help policy makers and program implementers to consider evidence regarding nutritional status among children of this specific group.

The objective of the study was to find the nutritional status of 6-59 month old Tharu children in Duruwa VDC of Dang District.

## METHODS

This study was descriptive cross-sectional conducted in Duruwa VDC that lies in mid-western development region, currently province 5 of Nepal. The study population was all 6-59 month old Tharu children living in concerned VDC. Written consent was obtained from the caretakers of the children. Those who denied participation in the project, seriously ill, or were not available at during the period of the study were excluded. The response rate was 100%.

A pre-coded questionnaire (partially adopted from Nepal Demographic Health Survey (NDHS) and was modified based on extensive literature review) was used to collect information and was administered to the caretakers. Anthropometric measurements (weight, height) were carried out to assess the nutritional status of children.

The samples of 189 children were selected by using simple random sampling technique for assessing nutritional status. Sample size estimation:


$$\begin{array}{c} \text{s}= \end{array}\frac{{\text{Z}}^{\underline{2}}}{}\frac{\times \text{p}\times \text{q}}{{\text{d}}^{2}}\begin{array}{c} =171 \end{array}$$

Where,

Z = 1.96 for Confidence Interval of 95%
p = Prevalence, 50% from previous

studies

q = 1- p
d= 0.075
Taking 10% non-response rate, the total sample size calculated was 189.

In households with more than one child of the age between 6-59 months, the younger child was chosen. Data collection period was between February and March, 2017.

For the analysis of Individual Dietary Diversity Score (IDDS), the questionnaire was classified with 12 food groups and we categorized IDDS on the following basis.^4^

- Low individual dietary diversity if consumed <5 groups
- Medium dietary diversity if consumed 5 to 8 groups per day
- High dietary diversity if consumed 9 to 12 groups per day

Based on 12 food groups, frequency of consumption was measured within 7 days and was calculated by adding the frequencies of consumption of all 12 food groups by the child. The score 1 is given for one food group eaten in one day from the 12 different food groups. Consumption of at least 5 or more food groups per day is considered as minimum dietary diversity met. The individual dietary diversity was determined by summing all the food groups consumed by the child. The lists of 12 foods are:

1. Cereals (Rice/bread/maize/lito/khichadi/pudding/ noodles etc.)
2. Root and tubers (potato/yam/githa/Pidalu etc.)
3. Fish and crab
4. Legumes and pulses (Bhatmas/chana/lentil/pea/ rahar etc.)
5. Milk and milk products (Milk/curd/yoghurt/paneer etc.)
6. Oil, Ghee, fat etc.
7. Sugar/honey etc.
8. Others (Tea/coffee/beverages)
9. Green leafy vegetables and other vegetables (Rayosaag/Spinach/Kholesaag/cauli flower/ cabbage/bottle guard/ash guard/pumpkin etc.)
10. Fruits (Papaya/banana/pomegranate/guava/ orange/bhogate etc.)
11. Meat (chicken/goat/buffalo/pig/duck/pigeon etc.)
12. Egg

Stadiometer was used to measure the height of children. The length of children below 2 years was measured by recombinant method and the height children of 2 years and above was measured in standing position.^5^

All clothes worn by children were removed and weight was measured by digital weight scale and read to the nearest 0.1 Kg. For children below two years of age and unable to stand by themselves, their weight was obtained from the difference between the weight of the mother as she holds her child and the weight of the mother alone. Age was determined from the birth certificate from the VDC and vaccination card based on availability.

The data was checked for completeness and consistency. The collected data was first organized, coded and entered into MS Excel and then into Statistical Package for Social Sciences (SPSS) version 20.0 and into World Health Organization (WHO) Anthro version 3.2.2. Response rate was 100% and there were no missing data. Descriptive analysis was carried out to describe the percentages and number distributions of the respondents by socio-demographic characteristics and other relevant variables in the study.

For IDDS cutoff point was used for categorizing in Low, medium and high dietary diversity.^4^ Anthropometric indices were calculated using reference medians recommended by WHO and classified according Z-score, based on WHO criteria.

Assuming the potential recall bias related to dietary intake and age of mother and children, to overcome this, cross questions were incorporated. Immunization card and birth registration card were cross-checked for the reported age.

Ethical clearance was obtained from Nepal Health Research Council. Verbal consent from parents/care taker of participants was obtained and the objective of the study was explained lucidly to them. Written consent was obtained from each caretaker of the participants before data collection.

## RESULTS

Total 189 participants were recruited in the study and they all were eligible, hence, included in the study. Out of them, 186 (98.4%) of household were Hindu and the rest 3 (1.6%) followed Christian religion. Those households having family members 5 or more than 5 were 144 (76.2%) whereas 45 (23.8%) of households had family members less than 5. The study shows that 61 (32.3%) of the household had an annual income less than 1 lakhs, 94 (49.7%) of the household had an annual income in range between 1 to 3 lakhs whereas 34 (18.0%) of the household earned more than 3 lakhs annually. The major occupation of the household as foreign employment with the highest percentage of 66 (34.9%) (Table 1).

**Table 1:** Socio-economic and demographic characteristics.

Variables Annual income	n (%)
Less than 1 lakhs	61 (32.3)
1 to 3 Lakhs	94 (49.7)
More than 3 lakhs	34 (18)
**Main occupation of house**	
Agriculture	59 (31.2)
Service	16 (8.5)
Labor	43 (22.8)
Business	5 (2.6)
Foreign Employment	66 (34.9)
**Family size**	
less than 5	45 (23.8)
5 or greater than 5	144 (76.2)

This survey showed that the breastfeeding was almost universal, 188 (99.5%) of mother breastfed their child. Feeding cow's milk as pre-lacteals was seen in this survey where 12 (6.3%) of total mothers fed cow's milk to their child before initiation of breastfeeding. Only 150 (79.4%) of household use packaged iodized salt. Almost all children 186 (98.4%) consumed Vitamin A. The result showed that most children had medium dietary diversity 118 (62.4%), followed by low dietary diversity 54 (24.6%), and very few had high dietary diversity 17 (9%) (Table 2).

**Table 2:** Child caring practices

Variables	n (%)
Breastfeeding status	
No	1 (0.5)
Yes	188 (99.5)
Time of initiation of breastfeeding	
Within 1 hour	143 (75.6)
Within 8 hours	17 (9)
After 24 hours	23 (12.2)
Don't remember	6 (3.2)
**Colostrum Feeding**	
Yes	185 (97.9)
No	1 (0.5)
Don't remember	3 (1.6)
**Feeding Prelacteals**	
Nothing	177 (93.7)
Cow's Milk	12 (6.3)
Exclusive breastfeeding	
Yes	160 (84.7)
No	29 (15.3)
**Feeding commercial milk**	
Yes	19 (10.1)
No	170 (89.9)
**Time of initiation of complementary food**	
4 months	2 (1.1)
5 months	15 (7.9)
6 month	167 (88.4)
7 month	5 (2.6)
**Type of salt consumption**	
Iodized	150 (79.4)
Non-iodized	39 (20.6)
**Vitamin A consumption**	
No	3 (1.6)
Yes	186 (98.4)
Place of first treatment	
Nearest Health post or Hospital	106 (56.1)
Pharmacy	36 (19)
Female Community Health Volunteer	41 (21.7)
DhamiJhakri or Religious Treatment	6 (3.2)
Individual dietary diversity score	
Low dietary diversity	54 (28.6)
Medium dietary Diversity	118 (62.4)
High dietary diversity	17 (9)

Out of 189 mothers, 23 (12.2%) of the mothers were illiterate. There were more adolescent mothers present in this study 143 (70.4%). The mean age at first pregnancy of the mothers in this survey was found to be 19.45±1.942 years. Only 160 (84.7%) of mothers were supplemented with iron tablets during their pregnancy while 15.3% were left out. The mean duration of intake of iron tablet was 177.12±56.015 days. 45 (23.8%) of children had low birth weight (less than 2.5 kg) and 19 (10.1%) of respondents didn't know the birth weight of their children. The mean birth spacing was 3.37±1.8 years (Table 3).

**Table 3:** Maternal and child characteristics.

Variables	Frequency (%)
**Maternal Characteristics**	
**Age group of mother**	
Less than 20	34 (18)
20–30	143 (75.7)
30 and above	12 (6.3)
**Mother's education**	
Illiterate	23 (12.2)
Primary level	90 (47.6)
Secondary level	58 (30.7)
Higher Secondary level or more	18 (9.5)
**Occupation of mother**	
House wife	154 (81.5)
Service	10 (5.3)
Labor	15 (7.9)
Business	10 (5.3)
**Age at first pregnancy**	
Less or equal to 20	133 (70.4)
Above 20	56 (29.6)
Intake of iron tablet	
No	29 (15.3)
Yes	160 (84.7)
**Child characteristics**	
Gender of Child	
Male	97 (51.3)
Female	92 (48.7)
**Birth weight of Child**	
Low birth weight	45 (23.8)
Normal birth weight	125 (66.1)
Don't Know	19 (10.1)
**Type of birth**	
Natural Birth	168 (88.9)
Caesarean Birth	21 (11.1)
**Age group (months)**	
6-11	21 (11.1)
12-23	67 (35.4)
24-35	33 (17.5)
36-47	45 (23.8)
48-59	23 (12.2)
**Birth order**	
1	104 (55)
2	64 (33.9)
3	16 (8.5)
4 and above	5 (2.6)

**Figure 1.**
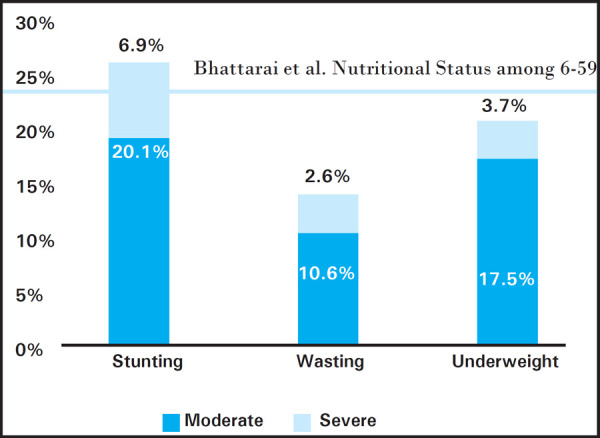
Present status of malnutrition in Tharu children of Duruwa VDC.

The overall magnitude of malnutrition were 51 (27%), 25 (13.2%) and 40 (21.2%) for stunting, wasting and underweight respectively (Figure I). Moreover, severe and moderate malnutrition were found among the stunting i.e. 38 (6.9%) and 13 (20.1%), wasting 20 (2.6%) and 5 (10.6%), and underweight i.e. 33 (3.7%) and 7 (17.5%), respectively (Figure 1).

The prevalence of stunting was seen more in females 26 (28.30%) than in males 25 (25.80%). The prevalence of those underweight was greater in males 23 (23.70%) than in females 17 (18.50%). The prevalence of wasting was very similar in males and females 13 (13.4%) and 12 (13.0%), respectively.

## DISCUSSION

Stunting and underweight among children of Duruwa VDC was found to be better than national average (stunting: 36%, underweight: 27%) whereas wasting was found to be worse than the country's national average (wasting: 10%). ^2^ The prevalence of wasting and underweight was found somehow similar with the nutritional status of under five children in Western Nepal where 15.1% were wasted and 20.2% were underweight respectively but the stunting was somehow lower in Duruwa VDC than in western Nepal.^6^ The inappropriate time of initiation of complementary feeding, outbreaks of diarrhea and other diseases may be the reason behind this. This may be due to low hygiene and sanitation in the community which may cause disease outbreak and parasitic infection in children causing wasting in children.

Children under five years of age are very vulnerable to the malnutrition. The children from the low income families are not breastfed properly and complementary foods are also not provided sufficiently so there is a high chance of malnutrition.^7^ During the survey, it was found that household having larger family use non-iodized salt as it is bought in bulk, making it relatively cheaper. So lack of iodine in both mother and child may hinder the adequate physical and mental development of child.^8^ The most of the mothers of Tharu community of Duruwa VDC were adolescent mother and had very less knowledge regarding feeding of children. Hence, long term inappropriate feeding behavior causes nutrient deficiency in children leading them towards malnutrition. Family having low income have very small allocation of budget for the food, the quality of the food might not be maintained, proper healthcare service might not maintained which may cause stunting in the children. Inadequate individual dietary diversity might be a risk factor for chronic malnutrition in Tharu children. Long term poor dietary diversity is likely to be reflected in stunting.^9^ Socio-economic indicator was solely based on income which measures limited facets of it. Study didn't measure actual calorie consumption and food consumption was based on food frequency questionnaire and individual dietary diversity scores only. There may be seasonal variation as the study was cross sectional. This study was conducted among limited population of specific Tharu community so it may not be generalizable to all population with different socio-economic settings.

## CONCLUSIONS

This study demonstrated that under-nutrition continues to be a serious problem in the Tharu community of Duruwa VDC. Interventions with integrated approach that improves overall socio-economic status, child care practices including maternal and child nutrition improvement is needed for overcoming the current nutritional problem. Also, to tackle the malnutrition related problem there is a great need to focus the attention of policy-makers on the nutritional status and government should prioritize nutrition related programs and implement it fully.
